# Precision breeding in agriculture and food systems in the United Kingdom

**DOI:** 10.1007/s11248-024-00397-7

**Published:** 2024-08-06

**Authors:** Oli Watson, Sadiye Hayta

**Affiliations:** 1 Capabilities in Academic Policy Engagement, London, UK; 2 Department of Crop Genetics, John Innes Centre, Norwich Research Park, Norwich, Norfolk, NR4 7UH UK

**Keywords:** Precision breeding, UK Precision Breeding Act, Gene editing, CRISPR

## Abstract

In recent years there have been major advances in precision breeding technologies, such as gene editing, that offer promising solutions to revolutionise global crop production and tackle the pressing issues in food systems. The UK has leading expertise in genomics, and research is already taking place to develop crops with improved resilience to climate change, resistance to disease and less reliance on chemical inputs. In March 2023, the Genetic Technology (Precision Breeding) Act received Royal Assent and passed into UK law. It provides a framework from which to build more proportionate regulations for plants and animals made using genetic technologies which contain genetic changes that could also arise through traditional breeding—known as ‘Precision Bred Organisms’. New legislation and the utilization of UK world-leading research could help to enhance the efficiency of breeding systems and enable the development of plants and animals that are healthier, better for the environment and more resilient to climate change.

## Introduction

According to estimates by the Food and Agriculture Organization (FAO), we will need to produce 60% more food by 2050 to feed a world population of over 9 billion. Food insecurity and low dietary quality cause public health problems. Increasing pressures on land and water, combined with climate change, poses a significant challenge in producing enough food sustainably and for a growing population. Addressing these complex challenges requires innovative approaches that not only boost crop productivity but also enhance nutritional content, resilience to climate change, and postharvest management.

Breeders have made use of our understanding of genetics for many years to create new varieties. They identify plants and animals that contain desirable characteristics and breed them together using traditional and modern breeding technologies to create a new generation that includes those characteristics. Plant breeding has helped to significantly improve yields and productivity over the last few decades (Steffen Noleppa 2021). However, traditional breeding can take several years to decades and may not be able to provide the pace of innovation needed to address food, climate and environmental challenges.

In recent years, significant strides have been made in the realm of genetics and new plant breeding technologies. This presents promising solutions to revolutionise global crop production and tackle the pressing issues in food systems.

Precision breeding is a term used to describe a set of technologies, including gene editing, that allow for the precise and efficient modification of an organism’s DNA to achieve desired traits. The key characteristic of precision breeding is that it can produce organisms with beneficial traits that could also occur through traditional breeding and natural processes, meaning it works within the existing genetic variability of a species. This means that breeders can make use of new technologies to introduce beneficial characteristics in a targeted way, where traditional breeding would otherwise take decades, or longer to achieve the same results.

This distinction is important because it sets precision breeding apart from genetically modified Organisms (GMOs), where modern techniques are used to insert functional DNA from an unrelated species into another species. GM, or genetic engineering, typically involves introducing genes from entirely different organisms (sometimes from different kingdoms) into the target organism to confer specific traits that may not naturally occur within the species.

Precision breeding, including technologies such as the CRISPR-Cas system and other site-directed nucleases (SDNs) like zinc finger nucleases (ZFNs) and transcription-activator-like effector nucleases (TALENs), are considered crucial components of precision breeding (Lusser et al. 2012; Zhu et al. 2017). These gene editing techniques allow for the precise modification of an organism’s DNA by adding, removing, or replacing genetic material at specific locations within the genome (Kamburova et al. 2017).

The process of gene editing typically involves inducing targeted DNA double-strand breaks (DSBs) at specific sites within a gene by using engineered nucleases. This prompts the cell’s DNA repair mechanisms, such as non-homologous end-joining (NHEJ) or homologous recombination (HR), to repair the DSBs, introducing the desired modifications at the target locus. This approach offers a high degree of precision, accuracy, and effectiveness, making it a valuable tool for breeding organisms with specific traits.

Site directed nucleases (SDNs) are proteins that can create DSBs at specific locations within a genome. The loci for strand cleavage are directed by nuclease-associated guides that are designed ex vivo to enable cleavage at a predetermined location. SDN-type genetic changes are grouped into three broad categories (SDN1-3) based on the genetic changes introduced at these targeted sites. All SDN-types can be used to produce multiple cleavage sites simultaneously.

SDN1 involves DNA cleavage followed by repair of the strand break by endogenous DNA mechanisms, usually NHEJ. NHEJ is a repair mechanism that often results in small insertions or deletions to the DNA sequence. These may alter the function of a protein or affect the action of non-protein coding regulatory sites.

SDN2 and SDN3 work in the same way but make use of an additional DNA template that is designed ex vivo. This template stimulates the cell to use an alternative repair pathway, Homology Directed Repair (HDR). HDR uses the template when repairing the cleaved DNA, precisely copying across the sequence from the template into the repaired strands. SDN2 is characterised by the use of DNA templates that introduce small, precise changes to the DNA. In contrast, SDN3 utilises large DNA templates that may introduce large-scale changes, such as perfect allelic replacement.

One notable advantage of gene editing, particularly with CRISPR-Cas technology, is its ability to produce transgene-free organisms. Transgenes are typically introduced to deliver the CRISPR-Cas system into the organism, but in subsequent generations, these transgenes can be segregated away through traditional breeding, leaving behind an organism with the desired genetic alterations but without foreign DNA.

Recent advances in sequencing technologies and bioinformatics tools have helped to gain deeper insights into crop genomes, facilitating the efficient and targeted modification of genes to impart desired traits.

### Recent legislative developments

The development of breeding has accelerated over the last decades through a greater understanding of genetics and the development of GM and now precision breeding technologies. GM technologies were seen as a step change because it could introduce genetic material from one species into another, different species (e.g., the Bt Delta microbial gene from Bacillus thuringiensis into maize—to control pest caterpillars). Most developed countries introduced legislation to check that organisms produced by this technology were safe and were defined legally as GMOs in the EU and in many other countries.

As technologies have developed, breeders are now able to develop organisms through precision breeding technologies that contain genetic changes that could have arisen through traditional breeding. This has challenged and prompted international debate on whether GMO regulations are adequate for organisms developed through these new precision breeding techniques. Many argue that regulations should be moving towards more of an outcome-based approach to assess the level of risk, and that current GMO systems are too onerous and prevent innovation and the realisation of benefits for the food system.

The international regulatory environment for products of genetic technologies is now rapidly evolving. Many countries such as Argentina, Japan, USA and Canada have already employed more proportionate regulatory systems for precision breeding, where the genetic changes could have also arisen through traditional breeding. Many others, including the EU, are now considering future regulatory changes (“New genomic techniques in biotechnology,” 2023).

England is moving in the same direction with a programme of regulatory reform. This is based on a broad scientific consensus that the existing GMO regulations are not appropriate or proportionate to the level of risk posed by precision breeding. The scientific advice is that precision bred organisms pose no greater risk than their traditionally bred counterparts.

The UK Government introduced new legislation in 2022 and 2023 to amend the regulation of GMOs in England. The aim of these new regulatory changes is to ensure that plants and animals developed using precision breeding technologies and food and feed produced from them, are regulated based on the science and proportionately to risk.

In April 2022, a new amending regulation to the Enviromental Protection Act (2022) was implemented to unlock research in plants and make it easier to carry out research field trials in England. Under this regulation, researchers are allowed to conduct field trials on ‘qualifying higher plants’ without the burden of going through the GMO (Genetically Modified Organisms) approval process. The key feature of this regulation is that researchers are required to notify the Department for Environment, Food and Rural Affairs (Defra) before conducting the field trials. By providing notification to Defra, researchers can proceed with their trials after 20 days without the need to undergo the more complex and time-consuming GMO approval process.

In March 2023, the Genetic Technology (Precision Breeding) Act received Royal Assent and passed into Law in England (this does not apply in Scotland, Wales and Northern Ireland). It provides a framework from which to build more proportionate regulations for plants and animals (excluding micro-organisms) made using modern biotechnology, which contain genetic changes that could also arise through traditional breeding—known as ‘Precision Bred Organisms’.

Under the Act, a new class of regulated product: ‘precision bred organisms’ has been introduced. Precision bred organisms will no longer be under the regulatory requirements of GMOs, and will be subject to new, more proportionate regulations.

The Act introduces two notification based regulatory systems for the environmental release and marketing of plants and animals. Before release into the environment (for research and non-marketing purposes), developers will need to send a ‘release notice’ notification to Defra before field trials can take place. This will be a self-certification that the organism is precision bred, and the field trial can take place 20 days after notification. The information collected will be published on a public register. This will subsume the process introduced in April 2022 for ‘qualifying higher plants.

For marketing purposes, developers will need to submit a ‘marketing notice’ notification to Defra and receive a confirmation from Defra Secretary of State that the organism is precision bred before it can be placed on the market. Under the Act, the UK’s independent scientific advisory committee ACRE (Advisory Committee on Releases to the Environment), is required to provide advice to the Secretary of State on the status of the organism. ACRE will assess information provided to Defra by the developer or breeder in the ‘marketing notice’. The information collected through this system will be published on a public register.

The definition of precision breeding aims to cover all organisms produced by modern biotechnology, but which contain genetic changes that could have occurred through traditional breeding. Drawing on scientific advice about the plasticity of genomes and the breadth of genetic changes that can be created through traditional breeding, the Act does not set defined limits or criteria. Guidance documents will help researchers and breeders to understand whether the organism is likely to be precision bred. In practice, this means that precision bred organisms may contain genetic changes arising through different site-directed nucleases (SDN) techniques. SDN1-3 are all acceptable so long as the genetic changes being introduced by these methods could have arisen in the existing gene pool through traditional processes.

This approach is in line with scientific evidence and advice because it focuses on regulating the end product of organisms to be released or marketed, rather than the process used to make them. The Act also establishes a regulatory system for the marketing of precision bred vertebrate animals to ensure animal welfare is safeguarded. Under the Act, anyone wishing to place a precision bred vertebrate animal on the market must submit an animal welfare declaration (with supporting evidence) which will be assessed by a new welfare advisory body. There may also be a requirement for ongoing reporting of health and welfare outcomes in precision bred animals and their qualifying progeny. Animals will still be regulated as GMOs until this system is in place.

For organisms used in the food or feed chain, the Act provides provisions for the Food Standards Agency (FSA) to establish a new science-based pre-market authorisation process for food and feed products. The FSA has responsibility to protect food safety and consumer confidence in food in England, Wales and Northern Ireland. The FSA will design a new framework that is more proportionate to the risk profile of precision bred food and feed products.

### Examples of precision breeding in UK

In many countries, including the UK, scientists have started to use precision breeding to accelerate the development of crops that are more nutritious crops, require fewer agrichemicals or are more resilient to climate change. Precision breeding in agricultural species has made remarkable progress in the past five years and research is already underway on many of the crops we eat every day, including wheat, tomatoes, brassicas, and pea.

Field trials play a crucial role in advancing agricultural research and development by providing valuable insights into how plants respond to real-world environmental conditions and agricultural systems. Recent legislative developments in the UK have provided agricultural research with the opportunity to fully exploit the potential offered by breakthroughs in technologies such as gene editing. By conducting field trials, researchers can better understand the practical implications of their innovations, leading to more informed and impactful agricultural advancements. Through these advancements, it is hoped that a sustainable and resilient future for agriculture and food systems can be achieved, ensuring food security for generations to come. Since the easing of regulation, there have been a number of trials for precision bred crops.

For example, researchers at Rothamsted Research successfully knocked out the asparagine synthase gene, TaASN2, in bread wheat genotype to reduce the concentration of free asparagine in the grain (Raffan et al. (2023). By doing so, they aimed to mitigate the conversion of free asparagine to acrylamide, a carcinogenic contaminant that forms during high-temperature food processing from wheat flour. Raffan et al. (2023) took the low asparagine edited wheat lines derived from the previous study and initiated the first field trial of gene-edited wheat lines in Europe.

Researchers at John Innes Centre (JIC) have used precision breeding to turn off a specific molecule in a plant’s genome and subsequently increase provitamin D3 in tomato plants (Li et al. 2022). By introducing specific genetic changes, these tomatoes can synthesize vitamin D, potentially enhancing the nutritional value of the fruit. This application demonstrates the potential of precision breeding to address nutritional deficiencies in crops and improve human health through enhanced dietary quality. The tomatoes were grown in an outside trial within the Norwich Research Park site within 20 days of submitting the notification. Using gene editing techniques, a JIC research team identified a key gene in wheat that can be used to introduce traits such as heat resilience while maintaining high yield. This discovery presents an opportunity to identify variations of the gene that can give wheat varieties resilience to climate change (Martín et al., 2021).

Tropic Bioscience, is a start-up company in Norwich Research Park in the UK, using precision breeding technologies to increase yield, extend shelf-life and improve natural disease resistance in banana. The company has developed a non-browning banana using precision breeding to reduce food wastage. This is now being authorised for use in other countries, such as the Philippines (https://tropic.bio/, 2023).

Researchers at The Sainsbury Laboratory have created a new tomato using precision breeding, named Tomelo, that is resistant to powdery mildew infection. Powdery mildew disease is one of the main reasons why UK tomato growers spray fungicides on their crops. This will help reduce the need to use fungicides and improve food production of UK tomatoes. It took less than 10 months to generate this resistant line, demonstrating the ability of precision breeding to make the breeding process more efficient and precise (Nekrasov et al. 2017).

The National Institute of Agriculture and Botany (NIAB) are also looking for ways to maintain or even increase wheat production while lowering the amount of fertilisers used—reducing the costs of production and working towards net zero (Milner et al. 2022). In this ongoing work, precision breeding techniques are being used to target genes that allows plants to continuing growing when nitrogen is a limiting factor.

Recent advances in precision breeding and crop transformation systems, in conjunction with the UK’s recent legislation changes, have all had a positive effect on research and development. The UK is at the leading edge of genetics and genomics research and encouraging the adoption and uptake of these new technologies will help to address food security challenges, climate change and biodiversity loss.

## Conclusions

In conclusion, precision breeding technologies hold significant potential to address food security, improve crop quality, and reduce postharvest losses. Examples like vitamin-d enhanced tomatoes, non-browning bananas, disease resistance showcases the transformative impact of precision breeding in agriculture. As precision breeding continues to advance, ongoing regulatory discussions will play a critical role in shaping the future of gene-edited crops and their integration into global food systems.

Moving to a more progressive, scientifically based approach to governing the use of products generated using genetic technologies could create significant economic opportunities for the UK.

As more gene edited crops undergo regulatory determination and approval processes in various countries, the agricultural landscape is likely to witness a transformation in crop improvement and sustainable food production.
